# Handwriting in children with Attention Deficient Hyperactive Disorder: role of graphology

**DOI:** 10.1186/s12887-019-1854-3

**Published:** 2019-12-10

**Authors:** Rony Cohen, Batia Cohen-Kroitoru, Ayelet Halevy, Sharon Aharoni, Irena Aizenberg, Avinoam Shuper

**Affiliations:** 1 Department of Pediatric Neurology and Epilepsy Center, Schneider Children’s Medical Center of Israel, Petach Tikva, Tikva, Israel; 2 Sackler Faculty of Medicine, Tel Aviv University, Tel Aviv, Israel; 3 Meuhedet Health Services, North District, Jerusalem, Israel; 4 Institute of Applied Graphology, Meitar, Jerusalem, Israel

**Keywords:** Handwriting, ADHD, Graphological analysis, Diagnosis

## Abstract

**Background:**

Handwriting difficulties are common in children with attention deficient hyperactive disorder (ADHD). The aim of our study was to find distinctive characteristics of handwriting in children with ADHD by using graphology to analyze physical characteristics and patterns, and to evaluate whether graphological analysis is an effective ADHD diagnostic tool for clinicians**.**

**Method:**

The cohort included 49 children aged 13–18 years attending a tertiary neurology and epilepsy center in 2016–2017; 22 had a previous DSM-IV/V diagnosis of ADHD. The children were asked to write a 10–12-line story in Hebrew on a blank sheet of paper with a blue pen over a 20-min period. The samples were analyzed by a licensed graphologist blinded to the clinical details of the children against a predetermined handwriting profile of individuals with ADHD. Each ADHD characteristic identified in each sample was accorded 1 point, up to a total of 15 points. Patients with a graphology score of 9–15 were considered to have ADHD.

**Results:**

There were 21 boys (43%) and 28 girls (57%) in the cohort; 15 boys (71.4%) and 7 girls (25%) had a DSM-IV/V diagnosis of ADHD. The mean graphology score was significantly higher in the children who had a DSM-IV/V diagnosis of ADHD than in the children who did not (9.61 + 3.49 vs. 5.79 + 4.01, *p* = 0.002, respectfully)**.** Using a score of 9 as the cutoff, in the girls, graphology had a specificity of 80% (95% CI 59.2–92.8) and a of sensitivity 71.4% for predicting ADHD**.** Corresponding values in the boys were 75.0 and 76.2%.

**Conclusion:**

The handwriting of children with ADHD has specific characteristics. Graphology may serve as a clinically useful tool in the diagnosis of ADHD.

## Background

Handwriting difficulties are common in children with attention deficient hyperactive disorder (ADHD) and have been associated with lower academic achievement and self-esteem [1–3]. Teachers report that the handwriting of both boys and girls with ADHD is immature, messy, and illegible**.** These findings may reflect poor motor skills and visual-motor integration, which are directly correlated with low handwriting legibility [2]. Furthermore, studies have shown that force, timing of agonist and antagonist muscles, and pen pressure are all weaker in children with ADHD [2, 4].

Tucha and Lange [5] studied the effect of methylphenidate on the quality and fluency of handwriting in children and adults with ADHD but did not evaluate changes in specific handwriting characteristics.

Graphology is an ancient discipline developed in China for purposes of analyzing the personality and behavior of individuals through the physical characteristics and patterns of their handwriting [6]. Following publication of the systematic theory of handwriting analysis by Ludwig Klages, a nineteenth century German philosopher and psychologist, the use of graphology for various purposes, including psychiatric research and evaluation, spread throughout Europe [7]. In 1942, the graphologist T.S. Lewiston and psychologist J. Zubin developed L-Z scales to objectively evaluate quantitative and qualitative handwriting elements, using statistical evidence to differentiate between handwriting of abnormal and normal personalities [8]. The scales made it possible for expert graphologists to identify relevant handwriting features in different languages and determine how they interact. Conclusions regarding either specifics or absolutes could not be reached on the basis of a single feature alone. It was the combination of several different features interacting in various ways that made it possible for clinicians to achieve a full and clear interpretation.

Since the mid-twentieth century, graphology has been applied in many settings: to find suitable employees, establish the authenticity of a signature or text, and establish the state of the author of a signature or a text, (e.g., drunk or anxious). It has also been used in court and during criminal investigations. Handwriting examinations are recognized clinical tools in psychiatry [7] for the diagnosis of suicide attempts [9] and severe major depressive disorder [10].

We hypothesized that children with ADHD might be distinguished by the types and number of abnormalities found in their handwriting. The aim of the present study was to use graphology to analyze the characteristics and patterns of handwriting in children diagnosed with ADHD compared to children without ADHD and to determine if graphology might serve as an objective auxiliary tool in the diagnosis of ADHD.

## Methods

### Participants

A total of 49 children aged 13 to 18 years were included in the cohort. The study group consisted of 22 children who presented at the clinics of Meuhedet Health Services (Northern District), one of the four publicly funded health maintenance organizations in Israel, between September 2016 and September 2017. All were diagnosed with ADHD by a senior pediatric neurologist using teacher and parent questionnaires followed by interviews with the child and at least one parent. The final diagnosis was based on the criteria of the Diagnostic and Statistical Manual of Mental Disorders, 4th (1994) or 5th (2013) edition (DSM IV/V) [11, 12]. The control group included 27 otherwise healthy children without ADHD who presented at the Pediatric Neurology and Epilepsy Center of Schneider Children’s Medical Center of Israel, a major tertiary pediatric hospital or at the Meuhedet Health Services (North District, Israel) clinics during the same time period because of a mild/moderate headache or simple viral infection. Inclusion criteria for all study participants were ability to speak and read Hebrew, attendance in a mainstream school, and absence of a severe learning disability or a psychiatric comorbidity (such as anxiety disorder, oppositional disorder, depressive disorder) requiring treatment or psychiatric intervention. None of the children had developmental coordination disorder. One child had Tourette syndrome with variable tics that did not need treatment.

The study was approved by Helsinki Committee of Rabin Medical Center. The parents or guardians of all children provided written informed consent prior to enrolment in the study.

### Procedure

Handwriting samples were collected from all participants. The children were given a blank sheet of paper and a blue pen and asked to write a story in Hebrew of 10–12 lines over a 20-min period. The papers were collected and submitted for analysis to a licensed forensic graphologist (B.C.-K.) who was blinded to the background and clinical data of the subjects.

### Graphology analysis

The graphologist established a predefined handwriting profile of individuals with ADHD based on graphology theory that handwriting can determine the type of personality and evaluated each sample accordingly. The profile was composed of 15 characteristics: text layout (spread out); margins (none or only one); line direction (never ascending); line, word and, letter spacing (all abnormal); nonconventional letters (many); handwriting slant (never to the right or ascending); deviation of handwriting (yes), letter size (abnormal, 3–4 cm); letter width (only wide or only narrow); continuity or flow connection (absent), shape of writing (never thread-shaped), writing speed (never slow), and strength of graphism (poor). In each sample, 1 point was accorded for every abnormal characteristic identified, yielding a graphology score ranging from 0 to 15. Patients with a score of 9 or more were considered to have ADHD.

### Statistical analysis

The graphology scores of the patients with and without ADHD were summarized as mean and standard deviation and compared between the groups, total and stratified by sex. Since scores showed a non-normal distribution, the non-parametric Mann-Whitney U test was used for data analyses. All tests were two-tailed, and the level of significance was set at *p* < 0.05. Chi-square test was used to compare categorical variables.

The diagnostic accuracy of the graphology evaluation was examined using receiver operating characteristic (ROC) curve analysis, which depicts sensitivity by 100% specificity for every possible cutoff score, with a resulting area under the curve (AUC) ranging from 0.5 (no better than chance) to 1 (perfect diagnostic accuracy). An AUC of 0.8 or higher suggests that an instrument can be considered a useful screening tool [13]. Sensitivity, specificity, positive/negative likelihood ratios (LR), and 95% confidence intervals (CIs) were calculated.

## Results

The total cohort included 22 boys (44.9%) and 27 girls (55.1%) of whom 14 boys (66.7%) and 7 girls (33.3%) had ADHD. Nine children (2 girls) had combined-type ADHD (43% of the total ADHD group). The subjects with and without a DSM IV/V diagnosis of ADHD were comparable in age distribution (mean ± SD, 15.53 ± 1.50 years vs. 14.87 ± 1.96 years, *p* = 0.204) and more likely to be male (66.7% vs. 25%, *p* = 0.002) .

**Fig. 1:**
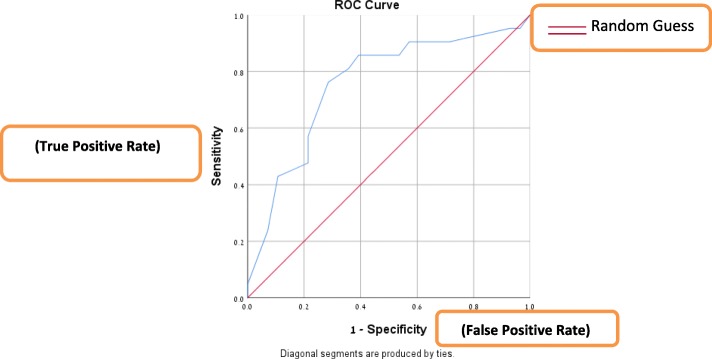
Receiver operating characteristic (ROC) curve for the performance of graphology in the diagnosis of ADHD

The children with a DSM-IV/V diagnosis of ADHD had a significantly higher mean graphology score than the children without ADHD (9.61 + 3.49 vs. 5.79 + 4.01, p = 0.002; Table 1) Analysis by sex revealed that among the girls, graphology scores were below 9 in 80% of the subjects without ADHD (95% CI 59.2–92.8) and 9 or higher in 71.5% of the subjects with a DSM-IV/V diagnosis of ADHD (95% CI 35.2, 93.5). Among the boys, the corresponding values were 62.5% (95% CI 29.5, 88.1) specificity and 78.6% (95% CI 53.1, 93.6) sensitivity. The total sensitivity of graphology to detect ADHD was 75% (95% CI 57.1, 88.1), and the total specificity, 76.2% (95% CI 55.4, 90.3) (Table 2). The AUC of the graphology scores was 0.756 (95% CI 0.635, 0.877), with a positive LR of 3.05 (95% CI 1.54, 6.04) and a negative LR of 0.32 (95% CI 0.140, 0.70) (Fig. 1). In females, the AUC was 0.757 (95%CI 0.568, 0.946), with a positive LR of 3.57 (95% CI 1.32, 9.65) and negative LR of 0.36 (95% CI 0.11–1.18). In males, the AUC was 0.705 (95% CI 0.506, 0.905), with a positive LR of 2.10 (95% CI 0.82, 5.34) and a negative LR of 0.34 (95% CI 0.11, 1.07).

**Table 1:** Mean scores by ADHD status and sex

	DSM	Mean	N	SD	*p* value Mann-Whitney U
F	Control	4.7500	20	4.05067	0.074
	ADHD	9.0000	7	4.47214	
	Total	5.8519	27	4.49533	
M	Control	8.3750	8	2.61520	0.158
	ADHD	9.9286	14	3.49647	
	Total	9.3636	22	3.23000	
Total	Control	5.7857	28	4.01255	0.002
	ADHD	9.6190	21	3.76133	
	Total	7.4286	49	4.31567	

*ADHD* attention-deficit-hyperactive disorder, *SD* standard deviat

**Table 2:** Criterion-validity of graphology-based ADHD score (0 as cutoff)

Gender	Graphology		DSM (gold standard)	
			Control	ADHD
Female	Negative	Count	16	2
		% within DSM (95%CI)	80.0 (specificity) (59.2–92.8)	28.6
	Positive	Count	4	5
		% within DSM (range)	20.0	71.4 (sensitivity) (35.2–93.5)
	Total	Count	20	7
		% within DSM	100.0	100.0
Male	Negative	Count	5	3
		% within DSM	62.5(specificity) (29.5–88.1)	21.4
	Positive	Count	3	11
		% within DSM	37.5	78.6 (sensitivity) (53.1–93.6)
	Total	Count	8	14
		% within DSM	100.0	100.0
Total	Negative	Count	21	5
		% within DSM (range)	75.0(specificity) (57.1–88.1)	23.8
	Positive	Count	7	16
		% within DSM (range)	25.0	76.2 (sensitivity) (55.4–90.3)
	Total	Count	28	21
		% within DSM	100.0	100.0

**Fig. 2:**
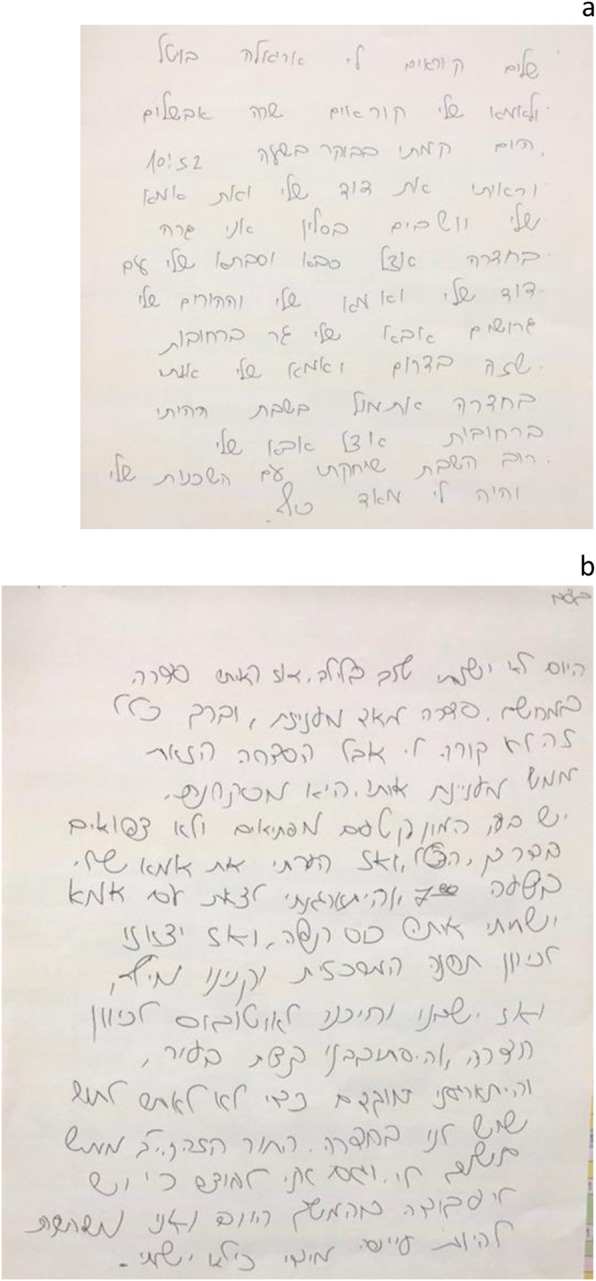
(**a**) Handwriting of a girl without ADHD (in Hebrew, right to left) (**b**) Handwriting of a girl with ADHD (in Hebrew, right to left). Note the abnormalities in the margins, text layout, and spacing between letters, words, and lines. In addition, there is inclination and deviation in the handwriting, and the letters are written unconventionally, with frills and a flow that are not part of written Hebrew.

On separate evaluation of the individual handwriting elements, the only significant feature in girls with ADHD was line direction (never horizontal or ascending) (*p* < 0.05; Fig. 2). Significant features in boys with ADHD were line spacing (abnormal) and writing speed (never slow) (p < 0.05).

## Discussion

Although it is well known that children with ADHD have handwriting difficulties and that their handwriting improves after treatment with methylphenidate [14], this is the first study to our knowledge to use graphology as a tool for evaluating ADHD. In support of our hypothesis, we found that graphology had a sensitivity of 75% and specificity of 76.2% for detecting ADHD.

Handwriting is a complex task requiring the integration of different components, including behavior (inattention, hyperactivity), motor planning, fine motor skills, and visual motor perception [15]. Lerer et al. [16] reported that the specific handwriting problems in children with ADHD were poor organization of written material within the space available, poor spacing within and between words, poor overall legibility, inconsistent letter size and shape, poor alignment, frequent erasures, frequent omissions of letters or words, letter inversions, poor rhythm and flow of writing, and slow speed. In our study, despite the good overall specificity and sensitivity of graphology, further analysis of the individual handwriting features yielded only a limited number of significant differences. Among the boys, writing speed was never slow in the subjects with ADHD compared to the control group who showed more variability. This finding differed from the study of Lerer et al. [15**]** but was in line with the results of Adi-Japha et al. [17] who reported that the kinematic manifestations of writing deficits in children with ADHD were a fast, inaccurate, and inefficient written product accompanied by high levels of axial pen pressure. The authors putatively explained this finding by the hyperactivity-associated hyperkinetic movements and lack of response inhibition characteristic of ADHD [18], leading the children to complete the task as quickly as possible. It is supported by the present study wherein there was no significant difference between the girls with and without ADHD, as females with ADHD are known to have fewer hyperactive/impulsive symptoms and more inattentive symptoms than males [19]. Other studies suggested that children with ADHD have a less appropriate speed of execution and more motor difficulties than children without ADHD [4, 20], but they did not distinguish the comorbid learning difficulties in ADHD that can cause lower writing speed [21]. Among the females, the handwriting of subjects with ADHD was never ascending compared to variable findings in the control group. Ascending/descending/fluctuating lines have been shown to be the most prevalent (53.6%) indicator of dysgraphia [22].

Thus, our study showed that handwriting evaluation by a graphologist can have incremental validity in terms of diagnostic accuracy in children with ADHD and can contribute to decision-making by the multidisciplinary team, especially in complicated cases Li-Tsang et al. [22] suggested that handwriting assessment can effectively distinguish children with ADHD or ADHD with learning disorders (ADHD-LD) from control subjects by the degree of variation in speed of writing or pen pressure.. Others have analyzed handwriting patterns in children with ADHD using computerized software [23]. The results showed that the ADHD group had poorer motor planning and execution skills and greater variability in motor control than the control subjects.

The main limitations of the present study are handwriting analysis by only a single graphologist, small sample size, and lack of data on other background factors such as socioeconomic class and parental education. Larger scale studies with multiple blinded graphologists are needed to corroborate our findings**.**

## Conclusion

The handwriting of children with ADHD has specific characteristics. The use of graphology for the diagnosis of medical disorders is a highly disputed branch of handwriting analysis. It is likely that handwriting problems in ADHD have less to do with the writing itself and more to do with factors related to motor control. Our study suggests that graphology is a promising potential auxiliary tool for use by clinicians in the diagnosis of ADHD.

## Supplementary information

**Additional file 1:** Files are related to Availability of data and material.

## Data Availability

All data generated or analyzed during this study are included in this published article (Additional file 1).

## Abbreviations

ADHD: attention deficit hyperactivity disorder
AUC: area under the curve
CI: confidence interval
DSM: Diagnostic and Statistical Manual of Mental Disorders
LR: likelihood ratio
ROC: receiver operating characteristic [curve]
