# Supplementary material for: Handwriting in children with Attention Deficient Hyperactive Disorder: role of graphology
Source: BMC Pediatr. 2019 Dec 10;19:484. doi: 10.1186/s12887-019-1854-3 (PMC6902409; doi:10.1186/s12887-019-1854-3)
Supplement: Supplementary file 1 — Additional file 1: Files are related to Availability of data and material. [file 12887_2019_1854_MOESM1_ESM.zip › 12887_2019_1854_MOESM1_ESM.docx]

Graphology analysis

**Text layout**

0=normal, 75% of the page

1=limited text

2= text spread out

3=text spread out only to upper/lower/side margin

**Margins**

0=left& margins and upper & lower margins

1=no left/right margins

2=no right/left margins

0=normal (if the space between two lines can fit for a line)

1-wide

2=narrow

3=unevenly spaced lines

**Words spacing**

0=normal (if the space between two words can fit for a letter)

1-wide

2=narrow

3=unevenly spaced words

**Letters spacing**

0=normal (1-2mm)

1-wide

2=narrow

3=unevenly spaced letters

**Handwriting inclination**

0= Straight lines

1= Ascending lines

2=Wavy lines

3= Descending lines

**Letter deviation**

0=right slant

1=upright

2=variable slant

3=left slant

**Size of handwriting**

0=normal 3-4 mm height

1=large size >5mm height

2=small size <2mm height

3=variable size

**Non-conventional letters**

0=conventional letters

1=non-conventional letters

**Width of letters**

0= normal 2.5 to 3.5

1= narrow letters <2mm

2=variable size letters

**Continuity or flow connection**

0= connected letters

1=disconnected, fragmented letters

2=variable flow connection

**Shape of letters**

1=concave shape

2=convex shape

3=angular shape

4=thread shape

**Speed of writing**

0= normal speed

1=fast speed

2=slow speed

**Strength of graphism**

0= poor intensity

1= Powerful strength
